# Developing implementation strategies for digital ICU diaries targeting ICU professionals: an implementation mapping approach

**DOI:** 10.1186/s43058-025-00767-0

**Published:** 2025-08-07

**Authors:** Carola M. A. Schol, Margo M. C. van Mol, Erwin Ista

**Affiliations:** 1 Department of Intensive Care, Erasmus MC, University Medical Centre Rotterdam, Research Intensive Care Ne-403, Rotterdam, CA 3000 The Netherlands; 2 Department of Internal Medicine, Section Nursing Science, Erasmus MC, University Medical Centre Rotterdam, Rotterdam, The Netherlands; 3 Department of Neonatal and Pediatric Intensive Care, Division of Pediatric Intensive Care, Erasmus MC-Sophia Children’s Hospital, University Medical Centre Rotterdam, Rotterdam, The Netherlands

**Keywords:** Intensive care unit, Digital ICU diary, Implementation mapping, Implementation strategy

## Abstract

**Background:**

Digital Intensive Care Unit (ICU) diaries can enhance the mental health of ICU survivors and their relatives by bridging memory gaps, improving understanding of ICU experiences, and providing emotional support. However, integrating digital diaries into routine ICU practice poses challenges, including privacy concerns, time constraints, and limited staff motivation. To date, no standardized implementation strategy exists. This study aimed to develop a theory-driven, stepwise implementation guide.

**Methods:**

A methodological, iterative developmental design was employed, guided by the Implementation Mapping (IM) framework. The first four IM steps structured the process. Step 1 involved a needs assessment using prior qualitative and quantitative studies, including focus groups and a survey with ICU professionals, to identify barriers and facilitators. In step 2, a collaborative session defined implementation outcomes, performance objectives, and key determinants for change. Step 3 involved selecting theory-based methods and implementation strategies using the taxonomy of behavior change methods. In step 4, these strategies were translated into practical actions and materials to support implementation.

**Results:**

The needs assessment (Step 1) identified 23 facilitators and 40 barriers, with survey findings confirming these insights. In step 2, fidelity, adoption, and sustainability were established as key implementation outcomes, with seven performance objectives linked to determinants such as knowledge, attitudes, and motivation. Step 3 resulted in the selection of twelve theory-based methods, including active learning, peer role modeling, and perspective-shifting, to inform strategy development. Finally, step 4 produced a structured implementation guide, adaptable to local contexts, designed to support ICU teams and champions in adopting and sustaining digital ICU diaries.

**Conclusions:**

This study demonstrates the value of Implementation Mapping for developing theory-driven implementation strategies for digital ICU diaries. The resulting guide systematically addresses barriers and facilitators, offering a scalable model for integrating digital health innovations into clinical practice.

**Supplementary Information:**

The online version contains supplementary material available at 10.1186/s43058-025-00767-0.

Contributions to the literature

- This study is the first to apply Implementation Mapping to systematically develop implementation strategies for digital ICU diaries.
- By integrating theory-driven methods, it offers a structured, replicable approach adaptable to other digital health interventions in ICU settings.
- The study highlights the critical role of champions in overcoming implementation barriers and sustaining innovations in ICU practice.
- Findings emphasize the importance of multi-level implementation strategies that align with the contextual determinants of healthcare settings.

## Background

Intensive care unit (ICU) diaries are designed to support the mental health of ICU survivors and their relatives [1, 2]. By documenting the patient’s medical condition and ICU experiences, these diaries may help fill memory gaps and enhance survivors’ understanding of their stay [3]. For relatives, ICU diaries may provide emotional and psychological support during and after a loved one’s critical illness [4]. ICU professionals also benefit by gaining a more holistic view of patients beyond the clinical setting. Additionally, entries from relatives offer valuable insights, enabling more personalized care and emotional support [5].

Digital ICU diaries offer several advantages over traditional paper-based ones, including improved accessibility, the ability to upload photographs, and collaborative input from patients, relatives, and professionals [6–8]. The digital diary used in this study is a secure, web-based application. ICU nurses introduce the diary to relatives shortly after ICU admission and provide an information envelope containing instructions and a unique activation code. When families choose to use the diary, they can activate it and invite others to contribute messages and images. Ownership of the diary remains with the family, who manage access rights. ICU staff can access the diary via a shortcut in the electronic medical record system using single sign-on. Each nurse has a personal dashboard and can access a specific patient’s diary via the activation code. Although the diary is not fully integrated into the electronic medical record, this setup enables secure and streamlined access without requiring a separate login. Computers with access to the medical record, and thus the diary portal, are available in each patient room.

Despite the benefits of digital ICU diaries, their implementation presents challenges, including perceived complex login processes, user-unfriendly interfaces, limited digital literacy, and skepticism regarding their added value over paper ICU diaries [7–9]. As a relatively new innovation, limited evidence exists to guide their successful implementation in ICU settings [10]. Educational strategies supported by diary champions—professionals who promote diary use and provide resources—have been used to implement paper ICU diaries. However, such strategies often lack a theoretical foundation to guide their design and evaluation [11, 12].

Implementation frameworks can systematically address barriers, ensuring the development of tailored, evidence-based, and scalable strategies suited to specific contexts, thereby increasing the likelihood of sustained success [10]. Grounding implementation strategies in theory is crucial, as theory explains how and why interventions achieve their intended outcomes, identifies modifiable determinants, and informs the selection of mechanisms to influence behavior [13]. Implementation Mapping (IM), a structured, five-step process, provides a systematic approach for planning, developing, and evaluating implementation strategies. By incorporating stakeholder input, behavioral theory, and empirical evidence, IM supports the adoption and integration of evidence-based interventions into practice, bridging the gap between research and implementation [14]. Therefore, we aimed to design a tailored strategy for implementing digital ICU diaries using the IM framework.

## Methods

### Design & setting

This study employed a methodological, iterative developmental design guided by the IM framework. It builds on two earlier studies that identified perceived barriers and facilitators to implementing digital ICU diaries at four hospitals in the Netherlands: one university medical center and three tertiary teaching hospitals [9, 15]. At the outset of the study, all four participating ICUs were still using paper-based diaries, although the level of implementation varied. In two hospitals, diary use was more embedded in daily practice and informally supported by aftercare nurses who routinely introduced and promoted the diary to families. In one of these hospitals, ICU nurses also contributed more frequently than in the other sites. In the remaining two ICUs, diary use was less consistent and dependent on individual initiative. None of the sites had formalized guidelines or integrated protocols regarding diary use. This contextual variation was considered during the tailoring of implementation strategies in the subsequent IM steps.

At that time, digital ICU diaries had not yet been adopted in daily practice. However, all four ICUs had committed to transitioning to digital formats and were actively engaged in implementation planning. Although shortcut access to the digital diary via the electronic medical record and single sign-on functionality were not yet operational during the needs assessment, the necessary technical infrastructure, such as computers in each patient room, was already in place, and preparations for diary integration had begun. Therefore, the level of implementation at the time of strategy development was pre-implementation. The strategy development process took place between May and September 2023. The target population consisted of ICU professionals, recognized as key stakeholders in both the implementation process and the eventual adoption of the digital diary. The study adhered to the Standards for Reporting Implementation Studies (StaRI) [16], as detailed in Supplemental File 1.

### Implementation mapping procedure

**Fig. 1:**
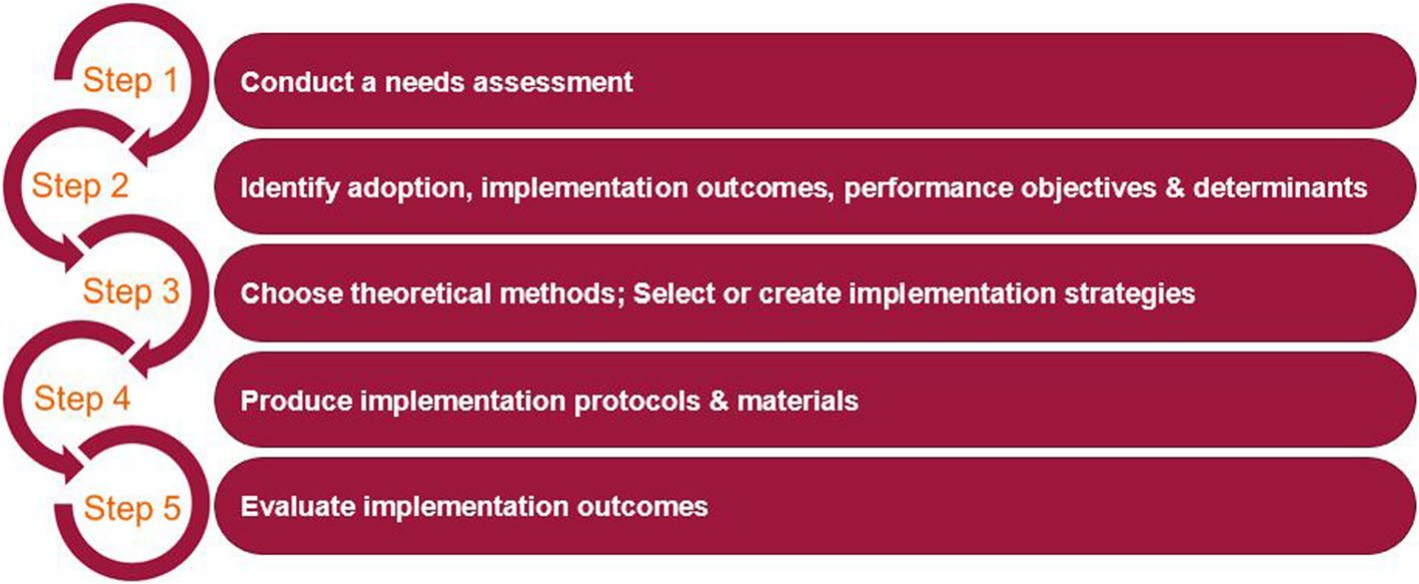
Five-step Implementation Mapping model [14]

We applied the five step IM protocol as described by Fernández et al. (2019), which combines principles of implementation science, theory-based behavior change, and stakeholder engagement to develop tailored implementation strategies [14]. In this study, we systematically conducted the first four IM steps. The fifth step, evaluation of implementation outcomes, is planned for a subsequent phase and is briefly outlined below. Figure 1 presents the five-step IM model. Throughout the process, we adhered to the iterative nature of IM by continuously refining objectives, methods, and materials in collaboration with stakeholders.

### Step 1. Conduct a needs assessment

This step involved identifying barriers and facilitators to implementing the digital ICU diary, guided by the updated Consolidated Framework for Implementation Research (CFIR) [17]. CFIR comprises five domains: (1) Innovation, (2) Outer setting, (3) Inner setting, (4) Characteristics of individuals, and (5) Process of implementation, encompassing 48 constructs relevant to the multifaceted aspects of implementation. We conducted five focus groups (FGs) with 32 ICU professionals from four hospitals, including ICU nurses, physicians, and social workers [9]. The semi-structured topic guide was developed using the updated CFIR and covered key implementation domains (Supplemental File 2). All FGs were audio-recorded, transcribed verbatim, and anonymized. Transcripts were analyzed using a mixed deductive–inductive approach, primarily employing directed content analysis [18]. Data were coded according to CFIR domains, with additional context-specific codes developed inductively. Two researchers coded independently and resolved discrepancies through discussion. Coding was conducted using ATLAS.ti© (v23.2.1). The findings of the FGs were used to develop a follow-up questionnaire, administered to 214 ICU professionals across the same hospitals [15]. This cross-sectional survey served to validate and prioritize the identified barriers and facilitators and to gather input on preferred implementation approaches. Together, reports of the qualitative FGs and the quantitative survey formed the foundation for the subsequent IM steps.

### Step 2. Identify adoption, implementation outcomes, performance objectives & determinants

In step two, we defined desired implementation outcomes using Proctor et al.’s taxonomy [19] and Damschroder et al.’s application of CFIR outcomes [20]. These frameworks provided a structured basis for establishing measurable goals. To operationalize these outcomes, we held a collaborative session to define performance objectives by systematically asking, “What actions must ICU professionals take to achieve these outcomes”? To understand the behaviors required, we identified modifiable determinants such as knowledge, beliefs and motivation [21], by posing the reflective question: “Why would an ICU professional choose to perform the desired behavior?” We then constructed matrices of change objectives, linking each performance objective to its relevant determinants. These matrices clarified what needed to change in each determinant to enable the desired behavior and served as the foundation for selecting appropriate implementation methods in the next step.

### Step 3. Choose theoretical methods; select or create implementation strategies

In step three, a second collaborative session was held to select theory- and evidence-based behavior change methods to address the determinants identified in step two. These methods, also known as behavior change techniques, are general strategies shown to influence one or more determinants of behavior [22, 23]. “Theory-based” indicates that each method is grounded in behavioral or social science theory, providing a rationale for its mechanism of action and anticipated impact [24]. The selection was guided by the taxonomy of behavior change methods [25], ensuring alignment with the determinants and parameters for effectiveness. Each method was mapped to its corresponding determinant using a structured matrix, which was then used to guide the selection and design of tailored implementation strategies, in a second collaborative session. We developed these strategies in line with the Expert Recommendations for Implementing Change (ERIC) framework [26]. The process was iterative and participatory, involving ongoing consultation with clinical experts and local stakeholders to ensure contextual relevance and feasibility. We developed a single, generic set of implementation strategies based on the overall results of the needs assessment. Tailoring to each ICU site occurred primarily in how the strategies were rolled out, particularly regarding the emphasis placed on specific strategies and the timing of their implementation. These local adaptations were informed by center-specific barriers and facilitators identified during the focus groups and surveys. This pragmatic approach preserved coherence in planning across sites while enhancing contextual relevance during implementation.

### Step 4. Produce implementation protocols & materials

In step four, we translated the selected strategies into a comprehensive set of actions and materials in line with Proctor et al.’s recommendations for supporting implementation, adoption, and long-term sustainability [27]. An interactive implementation guide was developed to support local implementation teams, with adaptable elements to address site-specific needs and contextual barriers. It included a step-by-step checklist, training plans, and customizable tools. Site-specific barriers identified in earlier steps were addressed through targeted strategies. We held a third collaborative meeting to refine the guide and used feedback from diary champions at each hospital to assess feasibility and inform final adjustments.

### Step 5. Evaluate implementation outcomes

Although this paper focuses on strategy development, we briefly outline the planned evaluation metrics, informed by Proctor et al.’s implementation outcomes framework [19]. In the next phase, the success of the implementation will be evaluated using the following outcomes: fidelity of the strategies, and fidelity, adoption, and sustainability of the digital diary. Fidelity will be assessed by examining the extent to which the strategies are executed as intended. This includes which strategies are applied in practice and whether diary use aligns with recommend practices. Adoption will be evaluated based on the proportion of digital diaries activated and the number of ICU professionals contributing entries. Sustainability will be assessed by examining the extent to which the digital diary becomes embedded in routine ICU workflows, for example through its integration into department-specific protocols and the use of automated reminders.

## Results

### Implementation mapping

The IM framework systematically guided the development of tailored strategies for implementing digital ICU diaries in four practical steps.

### Step 1. Needs assessment

**Fig. 2:**
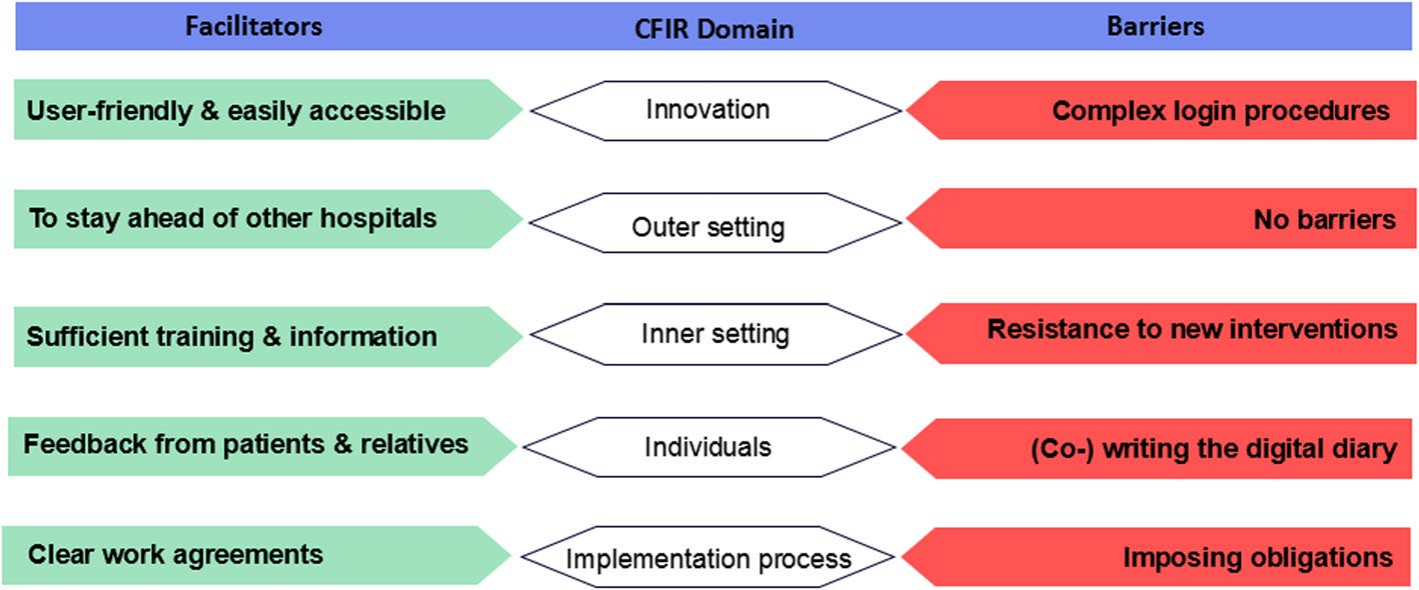
ICU professionals’ perceived barriers and facilitators to the implementation of a digital diary [9]

Thirty-two ICU professionals participated across five FGs in four hospitals. Median age was 41 [IQR 35–55], and 25 were women. Most participants were ICU nurses (*n* = 24; 75.0%), with additional participants including physicians (*n* = 4; 12.5%), medium care nurses (*n* = 2; 6.3%), one nurse practitioner (3.1%), and one social worker (3.1%). Each session lasted approximately 60 min. The FGs identified 23 facilitators across all five CFIR domains and 40 barriers across all domains, except the outer setting. Figure 2 provides an overview of the most frequently mentioned barriers and facilitators, categorized by domain. A complete list of all identified barriers and facilitators is provided in Supplemental File 3.

The follow-up questionnaire reinforced these findings. Key facilitators for implementation included seamless accessibility, motivated champions, and comprehensive education and information. The preferred dissemination method was direct instruction by team champions. Factors that supported providing digital diaries to relatives included understanding the diary’s utility and recognizing its added value [15].

### Step 2. Implementation outcomes, performance objectives and determinants

The selected implementation outcomes, as defined by Proctor et al. [19], included fidelity, adoption, and sustainability. These outcomes informed the development of seven performance objectives for ICU professionals, each linked to key determinants of change. Among these, knowledge emerged as the most foundational determinant, underpinning all performance objectives. Other determinants included beliefs, perceptions, attitudes, and motivation. While culture is not an individual determinant, it was recognized as a broader influencing factor. To operationalize these outcomes, we developed matrices linking each performance objective to one or more determinants, and then specified measurable change objectives. Table 1 provides a detailed overview of this step, including proposed measurement approaches and timing for each outcome.

**Table 1:** Outcomes, performance objective, determinants for change

Implementation outcomes	Performance objectives	Determinants for change	Measurement & timing
**Fidelity:** The digital ICU diary is successfully delivered to ICU professionals as intended	ICU professionals recognize the benefits of the digital diary and hold a positive view of its use	Knowledge Beliefs Perception	Interviews at Month 4, Post-Implementation survey (Likert scale) at Month 6
	ICU professionals possess sufficient knowledge about all aspects of the digital diary	Knowledge	Interviews at Month 4, Post-Implementation survey (Likert scale) at Month 6
	ICU professionals develop necessary skills to effectively use the digital diary	Knowledge Skills	Post-Implementation survey (Likert scale) at Month 6, observations champions
	ICU professionals remain unaffected by negative attitudes from colleagues regarding the digital diary	Attitude Knowledge Beliefs	Interviews at Month 4, Post-Implementation survey (Likert scale) at Month 6
**Adoption:** ICU professionals actively using the digital diary in their daily practice	ICU professionals inform relatives about the availability of the digital diary	Motivation Attitude Knowledge	Interviews at Month 4, Post-implementation survey at month 6, and audit of diary activation (Monthly)
	ICU professionals actively contribute to the digital diary by making regular entries	Knowledge Skills Motivation Beliefs Culture	System logs of entries (Monthly)
**Sustainability:** The digital diary becomes an integral part of ICU professionals’ daily responsibilities	ICU professionals consistently engage with the digital diary and integrate it into their workflows	Knowledge Motivation Beliefs Culture	Audit of workflow integration at month 6

### Step 3. Theory-based methods and implementation strategies

We identified twelve theory-based methods to address the determinants of change, using the taxonomy of behavior change methods. These included active learning, guided practice, perspective-shifting, modeling, direct experience, and feedback. Methods were grounded in behavioral theories such as Social Cognitive Theory (SCT) [28], and the Elaboration Likelihood Model of Persuasion [29], and were selected with attention to their parameters for effectiveness. While several methods were informed by SCT, particularly those targeting self-efficacy, modeling, and skill acquisition, we also drew on a broader theoretical base, including the Theory of Planned Behavior [30], Diffusion of Innovations [31], Goal-Setting Theory [32], Organizational Development Theories [33], and Theories of Learning [34]. These theories were matched to specific determinants such as motivation, cultural norms, and cognitive processing style, based on their relevance to ICU professionals’ implementation behaviors.

The methods were then translated into tailored implementation strategies, informed by the ERIC framework, and adapted to the ICU context. Although the strategy set was consistent across sites, local implementation was flexibly adapted. The emphasis and timing of specific strategies varied slightly between centers, depending on their existing practices and local barriers. For example, strategies targeting writing behavior, such as modeling and feedback, were implemented earlier or more frequently in ICUs where professional contributions to the diary were less established. Table 2 provides a comprehensive overview, mapping determinants to methods and strategies, and specifying the actor responsible for delivery, the timing of delivery, and the intended frequency. A more detailed version of Table 2, including additional behavioral determinants and strategy descriptions, is available in Supplemental File 4.

**Table 2:** ICU professionals’ determinants, theory-based methods, and implementation strategies

**Determinant for change**	**Theory-based methods**	**Definition**	**Parameters**	**Implementation strategies**	**Actor(s)**	**Timing/ Frequency**
**Knowledge**	**Active learning** Social cognitive Theory [28]	Encouraging learning from goal driven and activity-based experience	Time, information, and skills	Conduct educational meetings: o Organize a kickoff session at each center o Conduct educational sessions, interactive workshops, or brief on-the-job training sessions after the kickoff-session to ensure all team members are fully informed o Provide comprehensive training using diverse educational materials, such as videos, audio clips, and demos: o Disseminate information through multiple channels, including clinical lessons, newsletters, short info sessions on the ward, posters, and brief videos o Regularly reinforce information and education about the digital diary to keep colleagues’ knowledge up to date	Champions Team Leaders	Kickoff Month one Monthly refreshers
	**Elaboration** The Elaboration Likelihood Model of Persuasion [29]	Stimulating the learner to add meaning to the information that is processed	Messages should be clear, relevant, engaging, and include direct instructions to prompt active thinking in motivated, capable individuals	Make training dynamic: o Instruct colleagues on how to log in and use the digital diary through demo versions or on-the-job teaching o Use engaging materials such as presentations featuring videos, quotes, examples, and photos o Demonstrate how relatives can control who reads and contributes to the diary using demo versions o Guide staff on how to inform relatives about the digital diary o Highlight the writing suggestions and assistance tools integrated into the digital diary o Provide pocket cards with sample writing prompts and showcase examples of completed diary entries	Champions Team Leaders	Kickoff Month one
**Skills**	**Guided practice** Social Cognitive Theory [28]	Prompting individuals to rehearse and repeat the behavior various times, discuss the experience, and provide feedback	Subskill demonstration, instruction, and enactment with Individual feedback; supervision by an experienced person	o Organize hand-on practice sessions where champions demonstrate and encourage usage through demo versions o Offer opportunities for demonstrations and hands-on practice with champions during work o Ensure that sufficient written information is available for both professionals and relatives o Use role-playing exercises to practice offering the diary to relatives	Champions	Initial training Bi-weekly follow-up
**Attitude** **Beliefs** **Motivation** **Perception**	**Shifting perspective** Theories of Stigma and Discrimination [35]	Encouraging taking the perspective of the other	Initiation from the perspective of the learner	Create a supportive culture: o Inspire colleagues to use the digital diary o Discuss the benefits of the digital diary with colleagues o Emphasize the importance of writing and set a good example o Engage in conversations with colleagues about the digital diary o Promote the digital diary’s accessibility o Share success stories and positive testimonials from other hospitals	Local Implementation coordinator Team leaders Champions	Daily
	**Modeling** Social Cognitive Theory [28]; Diffusion of Innovations Theory [31]	Providing an appropriate model being reinforced for the desired action	Attention, remembrance, self-efficacy and skills, reinforcement of model; identification with model	Show role models from other hospitals: o Invite a nurse from another hospital who already works with the digital diary to attend the kickoff session, provide practical examples, and answer questions o Share videos of ICU nurses with experience using a digital diary explaining: ▪ Why the digital diary is important and its added value for professionals, patients, and their relatives ▪ Why nurses should both read and write in the digital diary ▪ How to introduce the digital diary to relatives and how to inform them about it	Champions External speaker(s)	Kickoff During training events
	**Arguments** Communication Persuasion Matrix [36]; Elaboration Likelihood Model of persuasion [29]	Using a set of one or more meaningful premises and a conclusion	For central processing of arguments, they need to be new to the message receiver	Raise awareness of the added value and benefits of the digital diary, as well as the importance of professional contributions through compelling arguments during the kickoff session, educational sessions or through workplace conversations	Local Implementation coordinator Champions	Kickoff Month one Daily
	**Feedback** Theories of Learning [34]; Goal-Setting Theory [32]	Providing feedback on progress and impact of performance or learning	Feedback needs to be individual, follow the behavior in time, and be specific	Provide monthly performance feedback on: ▪ The number of diaries activated in the past month ▪ The number of professionals contributing to the digital diary	Local Implementation coordinator Team leaders	Monthly
	**Direct experience** Theories of Learning [34]	Encouraging a process whereby knowledge is created through the interpretation of experience	Rewarding outcomes from the individual’s experience	Share testimonials from former ICU patients and their relatives through quotes, audio, or video’s during the kickoff session and educational sessions or in a monthly newsletter to highlight the benefits of the digital diary and professional contributions	Champions	Kickoff Monthly refreshers
**Culture**	**Participation** Diffusion of Innovations Theory [31]; Organizational Development Theories [33]	Assuring high level engagement of the participants’ group in problem solving, decision making, and change activities	Effective participation requires motivated, skilled participants and a facilitator who values their influence	Leadership support: o Ensure that team leaders actively support and promote the digital diary during start-of-day moments and end-of-day evaluations o Have team leaders emphasize the importance of the digital diary and encourage ICU professionals to engage with it	Team leaders	Daily
	**Facilitation** Social Cognitive Theory [28]	Creating an environment that makes the action easier or reduces barriers to action	Requires real changes in the environment	Integrate the digital diary into standard practices: o Integrate the diary into existing IT systems to simplify logging in (single sign-on) o Implement reminders in IT systems to encourage use o Make the diary a structured part of the admission process	IT Department	Pre- implementation
	**Resistance to social pressure** Theory of planned behavior [30]	Stimulating building skills for resistance to social pressure	Commitment to earlier intention; relating intended behavior to values; psychological inoculation against pressure	o Provide champions with coaching or training focused on managing colleagues’ resistance o Share success stories and positive testimonials from other hospitals, patients, and relatives to counter objections o Regularly highlight the number of activated diaries and the level of contributions by ICU professionals o Recognize and reward ICU professionals who consistently engage with the digital diary	External speaker(s) Champions Team leaders	Pre- implementation Initial training

### Step 4. Tailored implementation guide

The resulting implementation guide was based on the generic set of strategies and incorporated local adaptations derived from the site-specific barriers and facilitators identified in Step 1. It functioned as an implementation blueprint, a step-by-step workbook designed to support local project leaders and champions at each hospital in the preparation, implementation, and maintenance of digital ICU diaries. The guide was available in both print and digital formats; the digital version was provided as an editable document to allow ongoing adaptation to local circumstances as implementation progressed.

The guide was introduced during a dedicated champions day, a central training event during which ICU nurses identified as champions received in-depth training on the digital diary, including interactive sessions on responding to resistance and fostering team engagement. During this event, champions were also instructed on how to use the guide and encouraged to complete relevant sections tailored to their setting.

**Fig. 3:**
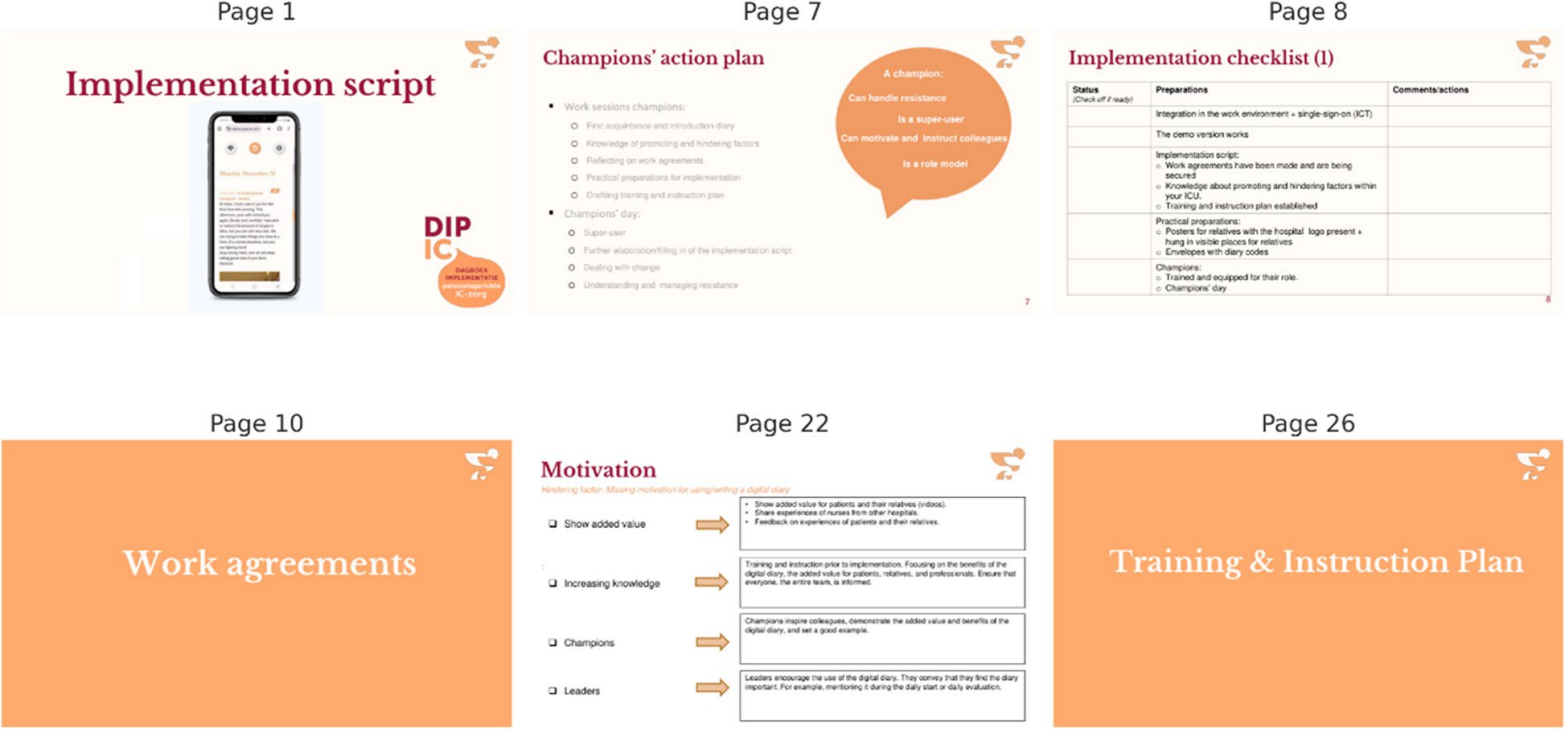
Implementation guide compilation

The content of the guide was customized to address hospital-specific barriers and provided practical strategies to overcome these challenges. Interactive in nature, it began with sections for documenting the roles and responsibilities of local project leaders, champions, and team leaders. It included an implementation checklist outlining key actions, such as testing the demo version, completing the guide, and preparing materials like posters for relatives and envelopes containing diary access codes. Additional sections allowed for documenting department-specific agreements, such as which patient categories would be offered digital diaries and the timing of their introduction to relatives. The guide also addressed hospital-identified barriers by offering practical suggestions and tips, while highlighting critical facilitators and key findings from the survey study. It concluded with a training and instruction plan, allowing champions to outline their plans for educational sessions, including content and delivery methods. Figure 3 presents selected pages from the guide, with the full version available in Supplemental File 5.

## Discussion

We systematically identified strategies for implementing digital diaries in the ICUs of four Dutch hospitals, using the IM framework. This methodological approach not only addressed practical implementation challenges in ICU settings but also contributed to the broader implementation science literature by demonstrating how theoretically informed strategies can enhance implementation efforts. The primary outcome of this study is an interactive, step-by-step guide, designed as a practical tool to prepare and support the implementation of digital diaries in ICU settings.

This study is among the first to specifically explore the implementation of digital ICU diaries using a systematic, theory-driven approach. Grounding implementation strategies in theory is essential, as theories help explain how and why interventions achieve their intended outcomes, identify modifiable determinants, and guide the selection of mechanisms to influence behavior and drive organizational change [13]. The IM framework proved helpful in designing and tailoring theory-based strategies to enhance the implementation, adoption, and sustainment of digital ICU diaries. This study also addresses a gap in the literature, where implementation strategies are often developed in an ad hoc manner without aligning with contextual determinants [37, 38]. As a result, many strategies fail to fit the specific contexts in which they are implemented, including the characteristics of the intervention, the healthcare setting, stakeholder preferences, and key implementation determinants [39].

Our findings highlight the critical role of champions as pivotal facilitators in implementation efforts [9, 15]. Given their influence, champions play a leading role in executing many of the implementation strategies we developed for digital diary adoption. These individuals are dedicated to promoting and driving the implementation process, overcoming resistance, and fostering engagement [26]. The literature consistently identifies champions as key drivers for successful change efforts in healthcare [40, 41], including ICU settings [11]. However, reliance on a single champion poses risks, as illness, turnover, excessive workload, or individual performance issues can hinder implementation efforts. Assigning multiple champions can help mitigate these risks and enhance sustainability [41]. For champions to be effective, they require targeted training, organizational support, and sufficient time to fulfill their roles. Their leadership, active engagement, and effective communication skills are crucial for ensuring the success of the implementation process [42].

Knowledge emerged as a fundamental determinant of change across all performance objectives. Educational strategies played a crucial role in bridging knowledge gaps and motivating ICU professionals to adopt digital diaries. To address both technical and behavioral barriers, tailored educational sessions, were delivered by champions, and supplemented by videos and interactive demo versions. Consistent with prior studies [11, 12, 43], our findings emphasize the importance of comprehensive education in successful implementation efforts. Resistance to change, often rooted in knowledge deficits, can be effectively mitigated through well-structured educational initiatives [44].

While this study primarily focused was on individual determinants influencing ICU professionals, organizational culture also plays a role in the adoption and sustainability of innovations [45]. Although, “culture” is a broad and sometimes ambiguous concept, its influence on team dynamics, leadership, and staff engagement is well-documented [46]. Resistance to change, often attributed to cultural factors, may instead stem from concerns about autonomy, workload, or the perceived value of the intervention [45]. Creating a supportive organizational environment – characterized by nursing leadership, shared decision-making, and active staff engagement – is essential for successful implementation [47]. The taxonomy of behavior change methods includes approaches for modifying social norms and organizational structures [25], and we categorized certain elements under the determinant of “culture”. However, our implementation strategies primarily focused on individual-level determinants rather than organizational influences. As a result, the cultural aspects influencing implementation success may remain underexplored. Future studies should explore these dynamics in greater depth to develop targeted interventions that address culture-specific barriers and facilitators, ultimately enhancing our understanding of the role of organizational dynamics and culture in broader implementation efforts.

Finally, it is essential to evaluate whether our implementation strategies have achieved the intended outcomes in terms of implementation, adoption, and sustainability. This can be accomplished by conducting the final IM step, “evaluate implementation outcomes” [14]. Implementation outcomes and corresponding performance objectives outline the specific actions required to successfully implement the intervention and serve as a foundation for developing evaluation instruments. Following the implementation of the digital ICU diary in four ICUs using the implementation guide developed in this study, we aim to assess the fidelity of both the implementation strategy and the digital diary, as well as its adoption and sustainability in clinical practice. This evaluation will involve observations, usage counts, multiple interviews with both champions and ICU professionals, and a questionnaire. Through this process, we aim to generate sufficient data to assess the effectiveness of our implementation strategy.

### Strengths and limitations

To our knowledge, this is the first study to apply the IM framework specifically to develop strategies for implementing digital ICU diaries. By leveraging this structured and systematic approach, the study provides a replicable model that can be adapted to other digital health interventions in ICU settings. A key strength lies in the two-phase needs assessment, which ensured a thorough, stakeholder-informed, and context-specific understanding of barriers and facilitators, providing a robust foundation for subsequent implementation steps. Additionally, the theory-driven selection of strategies enhances the likelihood of achieving meaningful changes in professional behavior [48]. However, this study also has some limitations. First, although the implementation strategies were informed by center-specific barriers and facilitators, we did not conduct a formal, structured decision-making process with local stakeholders to determine which strategies to emphasize. Instead, local adaptations occurred through interpretation of the qualitative and survey data by the research team. This may limit the transparency and replicability of how site-specific emphasis and timing were determined. Second, while the strategies primarily targeted ICU professionals as end-users, other key stakeholders, such as managers, policymakers, patients, and relatives, were not directly involved in the strategy development process, which may influence long-term implementation success. Finally, as the strategies were designed for four Dutch hospitals, generalizability to other healthcare settings may be limited.

## Conclusions

This study demonstrates the value of Implementation Mapping for systematically developing and adapting strategies to support the implementation of digital ICU diaries. By grounding these strategies in theory and aligning their delivery with key contextual determinants, we contribute to the growing field of implementation science and offer a replicable framework for integrating digital health interventions into routine clinical practice. While focused on ICU diaries, the insights gained also provide transferable guidance for advancing the adoption of evidence-based innovations in complex healthcare settings.

## Supplementary Information

Supplementary Material 1.

Supplementary Material 2.

Supplementary Material 3.

Supplementary Material 4.

Supplementary Material 5.

## Data Availability

The full dataset of the survey responses is available from the first author on reasonable request with requisite data use agreements in place.

## Abbreviations

CFIR: Consolidated Framework for Implementation Research
ERIC: Expert Recommendations for Implementing Change
ICU: Intensive Care Unit
IM: Implementation Mapping
