# Supplementary material for: Developing implementation strategies for digital ICU diaries targeting ICU professionals: an implementation mapping approach
Source: Implement Sci Commun. 2025 Aug 7;6:85. doi: 10.1186/s43058-025-00767-0 (PMC12330191; doi:10.1186/s43058-025-00767-0)
Supplement: Supplementary file 2 — Supplementary Material 2. [file 43058_2025_767_MOESM2_ESM.pdf]

Supplemental file 2. Topic list focus group interview ICU professionals

- *Current practice*
  - Is a paper diary currently utilized in your ICU?
  - Do professionals contribute to the paper diary?
  - For which types of ICU patients is the paper diary provided?
    - Could a digital diary function similarly for these patients?
    - Should there be additional criteria that patients or their family members must meet to use a digital diary?

- *CFIR domain 1. Innovation – digital diary*
  - Have you had the opportunity to review the digital diary?
    - If so: can you share what your first impression is?

- *CFIR domain 2. Outer setting*
  - Could the fact that the digital diary is used in other hospitals affect acceptance in your department?
  - Do experiences from professionals at another hospital regarding the digital diary influence your acceptance of the digital diary as a professional?
    - If yes, what would be the most effective method for sharing this information?

- *CFIR domain 3. Inner setting*
  - How would you characterize the culture within your intensive care unit in terms of its openness to change and new ideas?
  - Previous studies have identified barriers to implementing innovations or changes, such as high workload, time constraints and lack of motivation.
    - Do you also find these to be barriers when considering the implementation of the digital diary?
    - If so, do you have any suggestions on how to address these barriers effectively?
  - To what extent do the following individuals or groups promote or hinder the use of the digital diary?
    - Direct fellow ICU nurses
    - ICU physicians
    - Direct supervisors
    - ICU patients
    - Relatives

- ❖ *CFIR domain 4. Individuals*
  - What factors would make you willing to provide the digital diary to relatives of ICU patients?
  - As a professional, what factors might hinder you from offering the digital diary to relatives?

Professionals co-writing the digital diary:

- Apart from relatives contributing to and maintaining the diary, ICU professionals can also share brief messages in the diary.
  - What would encourage you to contribute to the digital diary in writing?
  - Are there any reasons that might discourage you from writing in the digital diary?

- ❖ *CFIR domain 5. Implementation process*
  - How do you believe the digital diary could be effectively introduced to the department?
  - What is the optimal method for conveying information or providing instructions about the digital diary?
    - What key information do you think is important for ICU professionals to understand about the digital diary?
    - Can the way information is presented influence your inclination to use the digital diary? If so, in what ways?
  - Are there any factors that may have a positive influence on the implementation and acceptance of the digital diary that have not yet been discussed in this interview?
  - Are there any factors that have a negative/impeding influence on the implementation and acceptance of the digital diary that have not already been mentioned in this interview
