# Supplementary material for: Developing implementation strategies for digital ICU diaries targeting ICU professionals: an implementation mapping approach
Source: Implement Sci Commun. 2025 Aug 7;6:85. doi: 10.1186/s43058-025-00767-0 (PMC12330191; doi:10.1186/s43058-025-00767-0)
Supplement: Supplementary file 3 — Supplementary Material 3. [file 43058_2025_767_MOESM3_ESM.pdf]

**Supplemental File 3. Facilitators and barriers categorized in CFIR**

| CFIR Domain | Construct | ICU professionals | Determinant |
|---|---|---|---|
| **1. Innovation domain** | Evidence-Base | Scientific research/evidence | Facilitator |
| | Relative Advantage | Digital is less accessible | Barrier |
| | | Digital is less personal | Barrier |
| | | Preference/advantages paper diary | Barrier |
| | | Privacy concerns | Barrier |
| | | Enhanced support for loved ones | Facilitator |
| | | A key component of person-centered care | Facilitator |
| | | Digital is easier | Facilitator |
| | | May reduce the number of phone calls | Facilitator |
| | | Digital is accessible anytime, anywhere | Facilitator |
| | Innovation Adaptability | The ability to write anonymously | Facilitator |
| | | Relatives decide on access professionals | Facilitator |
| | Innovation Complexity | Increase in administration | Barrier |
| | | Difficult to access the diary | Barrier |
| | | User-friendly & easily accessible | Facilitator |
| | Innovation Design | Photos directly uploaded in the diary | Facilitator |
| | Innovation Cost | Possible costs for relatives/patients | Barrier |
| **2. Outer setting domain** | External pressure | Not wanting to lag behind other hospitals. | Facilitator |

| CFIR Domain | Construct | ICU professionals | Determinant |
|---|---|---|---|
| **3. Inner setting domain** | Structural characteristics | Easily noticeable, good visibility | Facilitator |
| | Communications | Stays a part of the conversation | Facilitator |
| | Culture | Resistance to new interventions | Barrier |
| | Tension for change | Fits into today's (digital) society | Facilitator |
| | Compatibility | Already using a paper diary | Facilitator |
| | Relative Priority | Reduces time for direct patient care | Barrier |
| | Incentive Systems | Introduce a competitive aspect | Facilitator |
| | Materials & Equipment | High-quality info-material for relatives | Facilitator |
| | Access to Knowledge & Information | Sufficient training and information | Facilitator |
| | | Access to a demo version | Facilitator |
| | | Writing assistance and examples | Facilitator |
| | | There is already an overload of information | Barrier |
| **4. Individuals Domain**<br><br>**Sub-domain Roles** | Mid-level leaders | Encouraging and motivating manager. | Facilitator |
| | Implementation facilitators | Encouraging and motivating physicians | Facilitator |
| | | Experiences from other hospitals | Facilitator |
| | Implementation leads | Champions/ key-users | Facilitator |
| | Other implementation support | Feedback from patients and relatives | Facilitator |
| | Innovation Deliverers | Seeing no added value | Barrier |
| | | Handing over the diary to relatives needs more explanation | Barrier |
| | | Language barrier among relatives | Barrier |
| | | Inherent motivation | Facilitator |

| CFIR Domain | Construct | ICU professionals | Determinant |
|---|---|---|---|
| **4. Individuals Domain** | Innovation Recipients (Professionals) | Co-writing through professionals | Barrier |
| | | Diary is for personal use, not for professionals | Barrier |
| **Sub-domain Roles** | Innovation Recipients (patients/relatives) | Misplaced expectations of relatives | Barrier |
| | | Enthusiastic relatives | Facilitator |
| **Sub-domain** | Need | Knowledge of the benefits and importance to patients and relatives | Facilitator |
| **Characteristics** | Capability | Not everyone is digitally proficient enough | Barrier |
| | | Not knowing how it works | Barrier |
| | | Not knowing what to write. | Barrier |
| | | Knowing very well how the diary works | Facilitator |
| | Motivation | Lack of motivation for usage/writing. | Barrier |
| | | Negative past diary experience. | Barrier |
| **5. Implementation** | Assessing Needs | Imposing obligations on professionals | Barrier |
| **Process** | | Co-writing is optional for professionals | Facilitator |
| **Domain** | | Digital diary is the only diary offered | Facilitator |
| | | Both paper and digital diaries available | Facilitator |
| | | Able to have autonomy in work methods | Facilitator |
| | Planning | Work agreements should be clear | Facilitator |
| | | Thinking about integrating the diary in the work process from the very start | Facilitator |
| | Tailoring Strategies | Apply multiple implementation strategies | Facilitator |
| | Doing | The diary is put forward as a *fait accompli* | Facilitator |
| | Reflecting & Evaluating | Ensuring the diary remains in focus | Facilitator |
| | | Conduct regular evaluations | Facilitator |
| | Adapting | The diary is integrated in the admission protocol | Facilitator |
| | | Co-writing is a routine aspect of job duties | Facilitator |
