# Supplementary material for: Developing implementation strategies for digital ICU diaries targeting ICU professionals: an implementation mapping approach
Source: Implement Sci Commun. 2025 Aug 7;6:85. doi: 10.1186/s43058-025-00767-0 (PMC12330191; doi:10.1186/s43058-025-00767-0)
Supplement: Supplementary file 4 — Supplementary Material 4. [file 43058_2025_767_MOESM4_ESM.pdf]

Supplemental File 4

Table 2. (Complete) - ICU professionals' Determinants, Theory-Based Methods, and implementation strategies

Determinant for change	Theory-based methods	Definition	Parameters	Implementation strategies	Actor(s)	Timing/ Frequency
**Knowledge**	**Active learning**<br><br>Social cognitive Theory (28)	Encouraging learning from goal driven and activity-based experience.	Time, information, and skills.	Conduct educational meetings:<br><br>○ Organize a kickoff session at each center.<br>○ Conduct educational sessions, interactive workshops, or brief on-the-job training sessions after the kickoff-session to ensure all team members are fully informed.<br>○ Provide comprehensive training using diverse educational materials, such as videos, audio clips, and demos:<br>▪ Deliver complete information about the digital diary, highlighting its added value.<br>▪ Explain how to access the digital diary and emphasize its ease of use.<br>▪ Highlight the digital diary's safety and privacy aspects.<br>▪ Train staff how to introduce the digital diary to relatives and what information to communicate.<br>▪ Clarify the purpose, content, and value of professional contributions to the digital diary, providing concrete examples.<br>▪ Share experiences from other hospitals, preferably through a peer or experienced professional from another hospital.<br>▪ Showcase benefits of the digital diary for patients and their relatives, by including testimonials from former ICU patients and relatives.	Champions<br><br>Team Leaders	Kickoff<br><br>Month one<br><br>Monthly refreshers

				○ Disseminate information through multiple channels, including clinical lessons, newsletters, short info sessions on the ward, posters, and brief videos.<br>○ Regularly reinforce information and education about the digital diary to keep colleagues' knowledge up to date.		
	**Elaboration**<br><br>The Elaboration Likelihood Model of Persuasion (29)	Stimulating the learner to add meaning to the information that is processed.	Messages should be clear, relevant, engaging, and include direct instructions to prompt active thinking in motivated, capable individuals.	Make training dynamic:<br><br>○ Instruct colleagues on how to log in and use the digital diary through demo versions or on-the-job teaching.<br>○ Use engaging materials such as presentations featuring videos, quotes, examples, and photos.<br>○ Demonstrate how relatives can control who reads and contributes to the diary using demo versions.<br>○ Guide staff on how to inform relatives about the digital diary.<br>○ Highlight the writing suggestions and assistance tools integrated into the digital diary.<br>○ Provide pocket cards with sample writing prompts and showcase examples of completed diary entries	Champions<br><br>Team Leaders	Kickoff<br><br>Month one
**Skills**	**Guided practice**<br><br>Social Cognitive Theory (28)	Prompting individuals to rehearse and repeat the behavior various times, discuss the experience, and provide feedback.	Subskill demonstration, instruction, and enactment with Individual feedback; supervision by an experienced person.	○ Organize hand-on practice sessions where champions demonstrate and encourage usage through demo versions.<br>○ Offer opportunities for demonstrations and hands-on practice with champions during work.<br>○ Ensure that sufficient written information is available for both professionals and relatives.<br>○ Use role-playing exercises to practice offering the diary to relatives.	Champions	Initial training<br><br>Bi-weekly follow-up
**Attitude**<br><br>**Beliefs**<br><br>**Motivation**	**Shifting perspective**<br>Theories of Stigma and Discrimination (30)	Encouraging taking the perspective of the other.	Initiation from the perspective of the learner.	Create a supportive culture:<br><br>○ Inspire colleagues to use the digital diary.<br>○ Discuss the benefits of the digital diary with colleagues.<br>○ Emphasize the importance of writing and set a good example.<br>○ Engage in conversations with colleagues about the digital diary.	Local Implementation coordinator<br><br>Team leaders	Daily

**Perception**				○ Promote the digital diary's accessibility.<br>○ Share success stories and positive testimonials from other hospitals.	Champions	
	**Modeling**<br><br>Social Cognitive Theory(28); Diffusion of Innovations Theory (31)	Providing an appropriate model being reinforced for the desired action.	Attention, remembrance, self-efficacy and skills, reinforcement of model; identification with model.	Show role models from other hospitals:<br><br>○ Invite a nurse from another hospital who already works with the digital diary to attend the kickoff session, provide practical examples, and answer questions.<br>○ Share videos of ICU nurses with experience using a digital diary explaining:<br>▪ Why the digital diary is important and its added value for professionals, patients, and their relatives.<br>▪ Why nurses should both read and write in the digital diary.<br>▪ How to introduce the digital diary to relatives and how to inform them about it.	Champions<br><br>External speaker(s)	Kickoff<br><br>During training events
	**Arguments**<br>Communication Persuasion Matrix (32); Elaboration Likelihood Model of persuasion (29)	Using a set of one or more meaningful premises and a conclusion.	For central processing of arguments, they need to be new to the message receiver.	Raise awareness of the added value and benefits of the digital diary, as well as the importance of professional contributions through compelling arguments during the kickoff session, educational sessions or through workplace conversations.	Local Implementation coordinator<br><br>Champions	Kickoff<br><br>Month one<br><br>Daily
	**Feedback**<br><br>Theories of Learning (33); Goal-Setting Theory (34)	Giving information to individuals regarding the extent to which they are accomplishing learning or performance, or the extent to which	Feedback needs to be individual, follow the behavior in time, and be specific.	Provide monthly performance feedback on:<br><br>▪ The number of diaries activated in the past month.<br>▪ The number of professionals contributing to the digital diary.	Local Implementation coordinator<br><br>Team leaders	Monthly

		performance is having an impact.				
	**Direct experience** Theories of Learning (33);	Encouraging a process whereby knowledge is created through the interpretation of experience.	Rewarding outcomes from the individual's experience with the behavior or assurance that the individual can cope with and reframe negative outcomes.	Share testimonials from former ICU patients and their relatives through quotes, audio, or video's during the kickoff session and educational sessions or in a monthly newsletter to highlight the benefits of the digital diary and professional contributions.	Champions	Kickoff<br><br>Monthly refreshers
	**Belief selection**<br><br>Reasoned Action Approach (35)	Using messages designed to strengthen positive beliefs, weaken negative beliefs, and introduce new beliefs.	Requires investigation of the current attitudinal, normative and efficacy beliefs of the individual before choosing the beliefs on which to intervene.	Ensure ongoing promotion:<br><br>Continue emphasizing the benefits and added value of the digital diary through clinical teaching, written information, posters, team leaders, and champions.	Champions<br><br>Team leaders	Kickoff<br><br>Month 1<br><br>Monthly refreshers
**Culture**	**Participation** Diffusion of Innovations Theory (31);	Assuring high level engagement of the participants' group in problem solving, decision	Effective participation requires motivated, skilled participants and	Leadership support:<br><br>○ Ensure that team leaders actively support and promote the digital diary during start-of-day moments and end-of-day evaluations.	Team leaders	Daily

	Organizational Development Theories (36);	making, and change activities; with highest level being control by the participants' group.	a facilitator who values their influence	○ Have team leaders emphasize the importance of the digital diary and encourage ICU professionals to engage with it.		
	**Modeling**<br><br>Social Cognitive Theory(28); Diffusion of Innovations Theory (31)	Providing an appropriate model being reinforced for the desired action.	Appropriate models will vary by level.	Ensure that champions and team leaders inspire colleagues and lead by example.	Champions<br><br>Team leaders	Daily
	**Facilitation**<br><br>Social Cognitive Theory(28)	Creating an environment that makes the action easier or reduces barriers to action.	Requires real changes in the environment instead of in the perceptions of the environment.	Integrate the digital diary into standard practices:<br><br>○ Integrate the diary into existing IT systems to simplify logging in.<br>○ Implement reminders in IT systems to encourage use.<br>○ Make the diary a structured part of the admission process.	IT Department	Pre-implementation
	**Resistance to social pressure**<br><br>Theory of planned behavior (37)	Stimulating building skills for resistance to social pressure.	Commitment to earlier intention; relating intended behavior to values; psychological inoculation against pressure.	○ Provide champions with coaching or training focused on managing colleagues' resistance.<br>○ Share success stories and positive testimonials from other hospitals, patients, and relatives to counter objections.<br>○ Regularly highlight the number of activated diaries and the level of contributions by ICU professionals.<br>○ Recognize and reward ICU professionals who consistently engage with the digital diary.	External speaker(s)<br><br>Champions<br><br>Team leaders	Pre-implementation<br><br>Initial training

References

28. McAlister AL, Perry CL, Parcel GS. How individuals, environments, and health behaviors interact: Social cognitive theory. Health behavior and health education: Theory, research, and practice, 4th ed. San Francisco, CA, US: Jossey-Bass; 2008. p. 169-88.
29. Petty RE, Barden J, Wheeler SC. The Elaboration Likelihood Model of persuasion: Developing health promotions for sustained behavioral change. Emerging theories in health promotion practice and research, 2nd ed. Hoboken, NJ, US: Jossey-Bass/Wiley; 2009. p. 185-214.
30. Batson CD, Chang J, Orr R, Rowland J. Empathy, attitudes and action: Can feeling for a member of a stigmatized group motivate one to help the group. Personality and Social Psychology Bulletin. 2002;28(12):1656-66.
31. Rogers EM. Diffusion of Innovations 5th edition, ed. New York The Free Press; 2003. 776 p.
32. McGuire WJ. McGuire's Classic Input-Output Framework for Constructing Persuasive Messages. Public Communication Campaigns. 4 ed: SAGE Publications; 2013. p. 133-46.
33. Maibach EW, & Cotton, D. Moving people to behavior change: a staged social cognitive approach to message design. Designing Health Messages Approaches from communication theory and public health practice. Thousand Oaks, CA: Sage 1995. p. 41–64.
34. Kazdin AE. Behavior Modification in Applied Settings. 7 ed: Waveland Pr Inc; 2012.
35. Martin Fishbein IA. Predicting and Changing Behavior - The Reasoned Action Approach. 1 ed. New York Psychology Press; 2009.
36. Cummings T, Worley C. Organization Development & Change. 10th Edition2015.
37. Ajzen I. The theory of planned behavior. Organizational Behavior and Human Decision Processes. 1991;50(2):179-211.
