# Supplementary material for: Developing implementation strategies for digital ICU diaries targeting ICU professionals: an implementation mapping approach
Source: Implement Sci Commun. 2025 Aug 7;6:85. doi: 10.1186/s43058-025-00767-0 (PMC12330191; doi:10.1186/s43058-025-00767-0)
Supplement: Supplementary file 5 — Supplementary Material 5. [file 43058_2025_767_MOESM5_ESM.pdf]

Implementation script

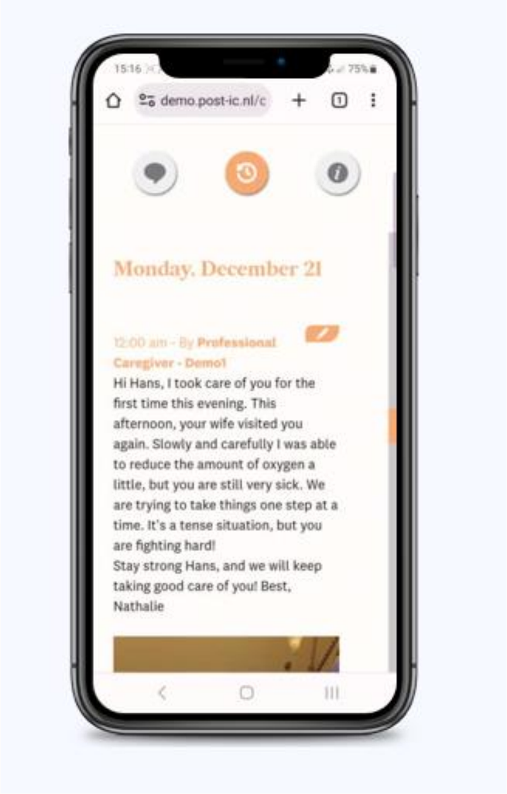

DIP IC
DAGBOEK IMPLEMENTATIE persoonsgerichte IC-zorg

The main outlines of the process

Focus implementation script

I	II	III	IV	V	VI
**Legal & privacy requirements**	**Drawing agreement**	**Setting up ICT**	**Promotion, introduction, instruction**	**Borging in the ICU work process**	**Evaluation**
		The diary will have a place in the work environment of employees, possibly also on other devices such as tablets.	The diary can be put into use. Of course, the nurses will first receive the necessary instructions.	The provision of the diary and the writing in the diary is secured within the activities of the ICU nurses.	Schedule meetings to evaluate the implementation process and experiences with the digital diary.

Timeline implementation

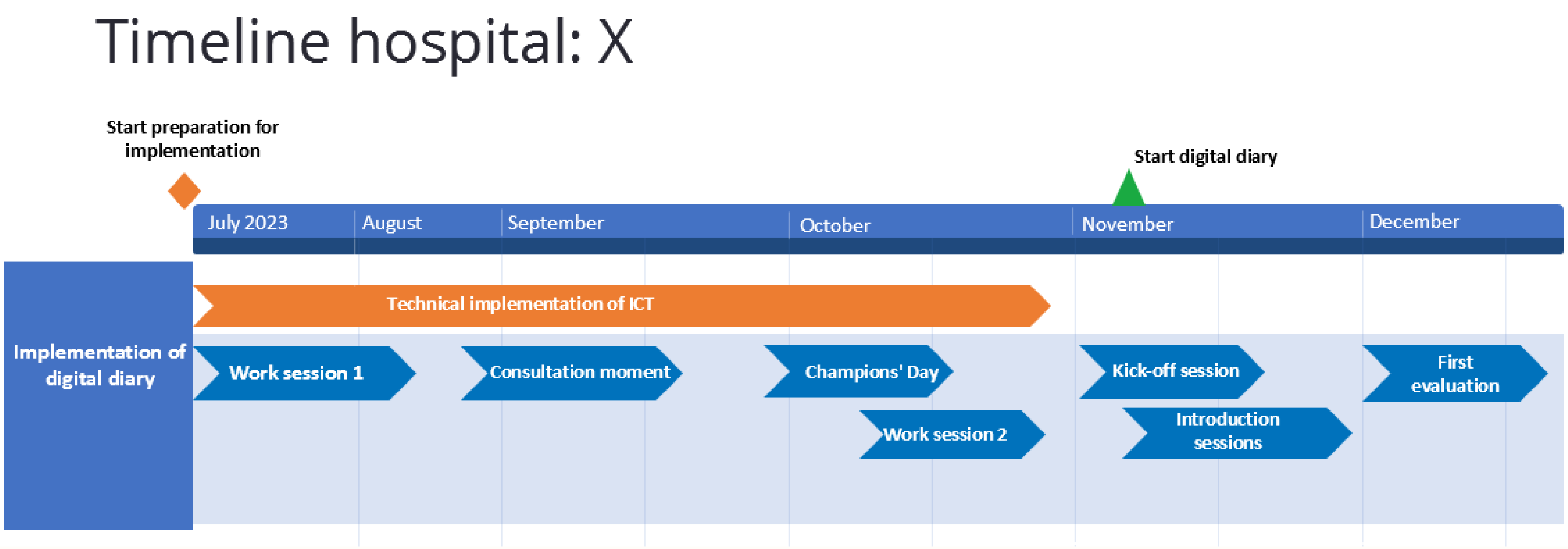

Local implementation leader

Who is the local implementation leader:

(name)………………………………………

It is important that there is someone who can act as a point of contact for all parties involved: coordinators, Games for Health, ICT, managers, researchers, etc.

Other tasks of the local implementation leader:

- ✓ Ensuring that agreed actions are carried out
- ✓ Monitoring whether implementation proceeds according to schedule

Champions

Wo are the champions:

- (Name)………………………………….
- (Name)………………………………….
- (Name)………………………………….
- (Name)………………………………….
- (Name)………………………………….
- (Name)………………………………….
- (Name)………………………………….
- (Name)………………………………….
- (Name)………………………………….

Champions advocate for the importance of the diary and take on a leadership role in introducing and maintaining the digital diary in the department.“

Some tasks include:

✓ Contributing ideas on procedures and work agreements
✓ Motivating and instructing colleagues
✓ Serving as role models
✓ Keeping the initiative in focus

Leaders

Who are the involved managers:

- (Name)……………………………………
- (Name)……………………………………
- (Name)……………………………………
- (Name)……………………………………

It is important for leaders to facilitate and encourage the use of the digital diary.

They can do this by:

- ✓ Bringing colleagues' attention to the digital diary.
- ✓ Emphasizing that they consider the digital diary important.

Champions' action plan

- Work sessions champions:
  - First acquintance and introduction diary
  - Knowledge of promoting and hindering factors
  - Reflecting on work agreements
  - Practical preparations for implementation
  - Draftiing training and instruction plan
- Champions' day:
  - Super-user
  - Further elaboration/filling in of the implementation script
  - Dealing with change
  - Understanding and managing resistance

A champion:

Can handle resistance

Is a super-user

Can motivate and instruct colleagues

Is a role model

Implementation checklist (1)

**Status**<br>*(Check off if ready)*	**Preparations**	**Comments/actions**
	Integration in the work environment + single-sign-on (ICT)	
	The demo version works	
	Implementation script:<br>○ Work agreements have been made and are being secured<br>○ Knowledge about promoting and hindering factors within your ICU.<br>○ Training and instruction plan established	
	Practical preparations:<br>○ Posters for relatives with the hospital logo present + hung in visible places for relatives<br>○ Envelopes with diary codes	
	Champions:<br>○ Trained and equipped for their role.<br>○ Champions' day	

Implementation checklist (2)

**Status**<br>*(Check off if ready)*	**Actual implementation and usage**	**Comments/actions**
	Kick-off:<br>o Date of the kick-off session<br>o Dates of introductory sessions for the rest of the team<br>o Information will be disseminated in various ways.	
	Leaders facilitate and encourage<br>Leaders convey that they consider the digital diary important	
	Ensure that the diary remains in the spotlight	
	Evaluate work agreements and adjust if necessary	
	Bring the digital diary into the workflow	
	Share feedback from patients and relatives regarding the digital diary with the team.	

Work agreements

Work agreements (1)

General

Where do we hang the posters for relatives?

Where do we hang the posters for professionals?

Where do we put the diary envelopes?

Work agreements (2)

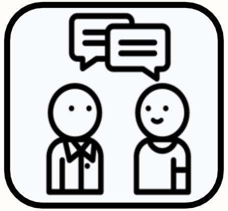

Offering the digital diary

To whom do we offer the digital diary?

Do we offer only the digital diary or also the paper version?

At what moment do we offer the digital diary?

Work agreements (3)

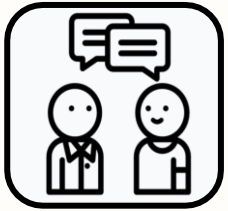

Offering the digital diary

Who offers the digital diary to the relatives?

How do I know that my colleague has offered the digital diary?

Work agreements (4)

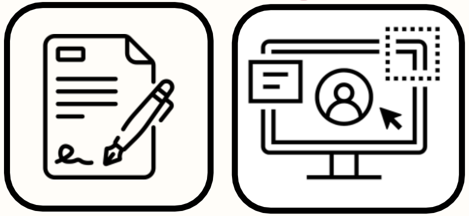

Consent form & access code

Who takes the signed consent form and who stores it or scans it in?

Who records the access code to the diary and where is this noted?

For example, in a visible location in the patient's file..

How do we know that the patient has a digital diary?

For example: By placing a magnet by the bed with 'Will you write for me?' Mention on the ICU Dashboard.

Work agreements (5)

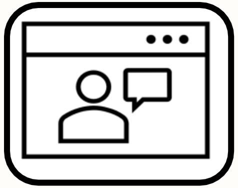

Writing in the digital diary

When is the digital diary written in?

Do we write under our own name or anonymously?

What do we write and what do we not write?

Agree not to share medical information + the diary is not a means of communication.

Work agreements (6)

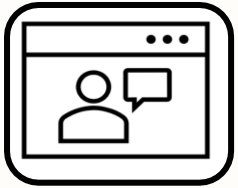

Photos in the digital diary

How do we handle photos in the digital diary?

If photos: What are the photos taken with?

Work agreements (7)

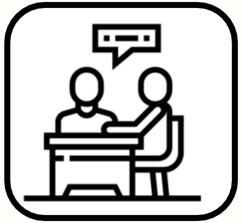

Conversation Follow-up

Is the digital diary used in the aftercare clinic?

Other work agreements

How do we ensure the safeguarding and evaluation?

How (often) do we monitor the usage?

Hindering factors & how to deal with them

Dealing with resistance

Hindering factor: Resistance & Dominant opposing voice

- ❑ Champions’ day → Champions are equipped through coaching sessions with knowledge and skills to deal with resistance and are strengthened in their role..

- ❑ Continuing to emphasize the advantages and added value of a digital diary in various ways. → For example: Include benefits and added value in training & instruction

- ❑ Example Role Champions → Champions continue to inspire colleagues and set a good example.

- ❑ Facilitating role of leaders → For example: Managers point out the digital diary during the daily start or daily evaluation. They encourage the use of the diary. They convey that they consider the diary important.

Easy access

Hindering factor: Difficulty logging in to access the diary

❑ Single sign-on, integration in digital workspace	Single sign-on will be implemented in each center. Accessible through the existing electronic patient file.
❑ Instruction and information	Adequate training and information, with videos and screenshots. Include in kick-off sessions and instructional moments for champions. Incorporate into work instructions.
❑ Demo version	Demonstration and practice with champions during work.
❑ Champions	Champions instruct colleagues and emphasize that the digital diary is easily accessible. For example: during clinical teaching moments, and while at work.

Professionals write in the digital diary

Hindering factor: Professionals contributing to diary entries

❑ Show added value	⇨	• Demonstrating added value for patients and their relatives (videos).<br>• Sharing experiences of nurses from other hospitals.<br>• Providing feedback on the experiences of patients and relatives.
❑ Increasing knowledge	⇨	Clinical lecture or interactive workshop prior to implementation. Focusing on the why of writing and also 'what do I write'.<br>Kick-off session per center including a nurse from another hospital.<br>Continuing education through champions (to reach everyone). Also mention that it is possible to write anonymously.
❑ Skills	⇨	Clear instructions written out. Training using a demo version.<br>Examples of what to write shown in clinical lessons, cards with writing examples.<br>Writing suggestions and writing assistance in the digital diary..
❑ Champions	⇨	Champions convey that writing is important and set a good example.<br>Champions engage in conversations with colleagues.

Motivation

Hindering factor: Missing motivation for using/writing a digital diary

❑ Show added value

- Show added value for patients and their relatives (videos).
- Share experiences of nurses from other hospitals.
- Feedback on experiences of patients and their relatives.

❑ Increasing knowledge

Training and instruction prior to implementation. Focusing on the benefits of the digital diary, the added value for patients, relatives, and professionals. Ensure that everyone, the entire team, is informed.

❑ Champions

Champions inspire colleagues, demonstrate the added value and benefits of the digital diary, and set a good example.

❑ Leaders

Leaders encourage the use of the digital diary. They convey that they find the diary important. For example, mentioning it during the daily start or daily evaluation.

Facilitating factors

Facilitating factors

	Top 10 facilitating factors Hospital X	Action (what/who)	Status
1	Champions		
2	Contribution to work agreements/ Evaluation		
3	Training and information digital diary		
4	Motivating role of leaders		
5	Stimulating role of physicians		
6	Sharing benefits, importance for patients and relatives		
7	User-friendly & easily accessible		
8	Possibly fewer phone calls from relatives		
9	Nurses are intrinsically motivated / see the value of the digital diary		
10	Consider embedding it into the work process from the implementation stage.		

Good to know

(Outcomes of questionnaire research)

In the implementation of the digital diary, it is important that:

Bevorderende factoren

- Logging in is easy.
- Champions are enthusiastic and motivate colleagues.
- Complete information is provided about all the ins and outs regarding the digital diary (including added value and privacy aspects).
- Managers and physicians play a facilitating role.
- Experiences and methods of nurses from other hospitals are shared.
- IC professionals have a say in the work agreements that are made.
- The diary is brought to attention in various ways.

- *During the use of the digital diary, it is important that:*
- Experiences of relatives and patients are communicated back to the team.
- Work agreements and procedures are regularly evaluated with the team.

Training & Instruction Plan

Training and Instruction Plan Team (1)

Step-by-step plan:

- Kick-off session
  - As many people from the team as possible.

- Champions instruct other team members in the following ways:
  - ……………………………………………………………………………………
  - ……………………………………………………………………………………
  - ……………………………………………………………………………………
  - ……………………………………………………………………………………

Ways for information dissemination:

- ✓ Training during work
- ✓ Demo version
- ✓ Clinical lessons / instruction moments
- ✓ Meeting, consultation moments
- ✓ Written information in the department
- ✓ Information via work email
- ✓ Videos

Ps: keep track of who has been trained

Training and Instruction Plan Team (2)

- The following matters must be addressed in the training & instruction:
  - ....
  - ....
  - ....
  - ....
  - ....
  - ....
  - ....
  - ....

What is important:

- ✓ Benefits/added value
- ✓ Work agreements
- ✓ How to log in/access
- ✓ Experiences from other hospitals
- ✓ Experiences of patients and relatives
- ✓ Write examples

DIP IC

Dagboek Implementatie
als opmaat naar
geïntegreerde interventies
in **persoonsgerichte IC-zorg**
